# Progesterone receptor variation and risk of ovarian cancer is limited to the invasive endometrioid subtype: results from the ovarian cancer association consortium pooled analysis

**DOI:** 10.1038/sj.bjc.6604170

**Published:** 2008-01-22

**Authors:** C L Pearce, A H Wu, S A Gayther, A E Bale, P A Beck, J Beesley, S Chanock, D W Cramer, R DiCioccio, R Edwards, Z S Fredericksen, M Garcia-Closas, E L Goode, A C Green, L C Hartmann, E Hogdall, S K Kjær, J Lissowska, V McGuire, F Modugno, K Moysich, R B Ness, S J Ramus, H A Risch, T A Sellers, H Song, D O Stram, K L Terry, P M Webb, D C Whiteman, A S Whittemore, W Zheng, P D P Pharoah, G Chenevix-Trench, M C Pike, J Schildkraut, A Berchuck

**Affiliations:** 1 Department of Preventive Medicine, Keck School of Medicine, University of Southern California Norris Comprehensive Cancer Center, 1441 Eastlake Avenue, Room 4415A, Los Angeles, CA 90089, USA; 2 Translational Research Laboratories, Windeyer Institute, University College London, 46 Cleveland Street, London W1T 4JF, UK; 3 Department of Genetics, Yale University School of Medicine, 333 Cedar Street, SHM I-321, New Haven, CT 06510, USA; 4 The Queensland Institute of Medical Research, Post Office Royal Brisbane Hospital, Herston, Brisbane QLD 4029, Australia; 5 Center for Cancer Research, National Cancer Institute, National Institutes of Health, MSC 4605, 8717 Grovemont Circle, Gaithersburg, MD 20892-4605, USA; 6 Obstetrics and Gynecology Epidemiology Center, Brigham and Women's Hospital, 221 Longwood, Boston, MA 02115, USA; 7 Department of Cancer Genetics, Roswell Park Cancer Institute, Buffalo, NY 14263, USA; 8 Magee-Womens Research Institute, University of Pittsburgh, 204 Craft Avenue, Pittsburgh, PA 15213, USA; 9 Department of Health Sciences Research, Mayo Clinic College of Medicine, 200 First Street SW, Rochester, MN 55905, USA; 10 Division of Cancer Genetics and Epidemiology, National Cancer Institute, National Institutes of Health, 6120 Executive Boulevard, Room 5014, Rockville, MD 20852-7234, USA; 11 Department of Virus, Hormones and Cancer, Institute of Cancer Epidemiology, Danish Cancer Society, Rigshospitalet Strandboulevarden 49, Copenhagen DK-2100, Denmark; 12 Department of Cancer Epidemiology and Prevention, Cancer Center and M Sklodowska-Curie Institute of Oncology, Roentgena 5, Warszawa 02-781, Poland; 13 Division of Epidemiology and Biostatistics, Department of Health Research and Policy, Stanford University School of Medicine, Stanford, CA 94305, USA; 14 Department of Epidemiology and University of Pittsburgh Cancer Institute, Pittsburgh, PA, USA; 15 Department of Epidemiology and Public Health, Yale University School of Medicine, 60 College Street, PO Box 208034, New Haven, CT 06520-8034, USA; 16 Division of Cancer Prevention & Control, H Lee Moffitt Cancer Center, 12902 Magnolia Drive, Tampa, FL 33612, USA; 17 CR-UK Department of Oncology, Strangeways Research Laboratory, University of Cambridge, Worts Causeway, Cambridge CB1 8RN, UK; 18 Department of Pathology, University of Arizona Medical College, 1501 North Campbell Avenue, Tucson, AZ 85724, USA; 19 Division of Preventive Medicine, The Duke Comprehensive Cancer Center, Durham, NC 27710, USA

**Keywords:** ovarian cancer, progesterone receptor, SNPs, PROGINS, pooled analyses, endometrioid ovarian cancer

## Abstract

There is evidence that progesterone plays a role in the aetiology of invasive epithelial ovarian cancer. Therefore, genes involved in pathways that regulate progesterone may be candidates for susceptibility to this disease. Previous studies have suggested that genetic variants in the progesterone receptor gene (PGR) may be associated with ovarian cancer risk, although results have been inconsistent. We have established an international consortium to pool resources and data from many ovarian cancer case–control studies in an effort to identify variants that influence risk. In this study, three PGR single nucleotide polymorphisms (SNPs), for which previous data have suggested they affect ovarian cancer risk, were examined. These were +331 C/T (rs10895068), PROGINS (rs1042838), and a 3′ variant (rs608995). A total of 4788 ovarian cancer cases and 7614 controls from 12 case–control studies were included in this analysis. Unconditional logistic regression was used to model the association between each SNP and ovarian cancer risk and two-sided *P*-values are reported. Overall, risk of ovarian cancer was not associated with any of the three variants studied. However, in histopathological subtype analyses, we found a statistically significant association between risk of endometrioid ovarian cancer and the PROGINS allele (*n*=651, OR=1.17, 95% CI=1.01–1.36, *P*=0.036). We also observed borderline evidence of an association between risk of endometrioid ovarian cancer and the +331C/T variant (*n*=725 cases; OR=0.80, 95% CI 0.62–1.04, *P*=0.100). These data suggest that while these three variants in the PGR are not associated with ovarian cancer overall, the PROGINS variant may play a modest role in risk of endometrioid ovarian cancer.

Several lines of evidence support a role for progesterone in the aetiology of ovarian cancer (Risch, 1998). Epidemiological studies have consistently shown a significant protective effect of parity. The protective effect increases steadily with each birth and pregnancy is associated with high progesterone levels (Hartge *et al*, 1989; Cooper *et al*, 1999; Titus-Ernstoff *et al*, 2001; Whiteman *et al*, 2003; Pike *et al*, 2004). In the third trimester, progesterone levels are some 10–15 times higher than in the luteal phase of the normal menstrual cycle. Oral contraceptives are also protective against ovarian cancer (Study, 1987; Rosenblatt *et al*, 1992, 1994; Ness *et al*, 2000; Royar *et al*, 2001; Schildkraut *et al*, 2002) and use of progestin-containing oral contraceptives increases average circulating progesterone levels to 9.2 ngm^−1^ compared to ∼3.5 ngm^−1^ during the normal menstrual cycle (Norman and Litwack, 1997). The protective effect of oral contraceptives per month of use is less than the protection from births, in line with the concentrations of progesterone. There is also some evidence that oral contraceptives with higher progestin content afford more protection against ovarian cancer (Schildkraut *et al*, 2002).

Animal models and *in vitro* data also suggest that progesterone has a significant influence on the ovary and on ovarian cancer. Studies in macaques suggest an apoptotic effect of progestins on the surface of the ovary (Rodriguez *et al*, 1998). *In vitro* treatment of both benign and malignant ovarian tumour cells with progestins results in an antiproliferative response (Zhou *et al*, 2002).

Progesterone binds to the progesterone receptor (PR) to initiate signalling. Two progesterone receptor isoforms (PR-A, PR-B) are encoded by a single gene (PGR). Except for a 164 amino-acid sequence at the N-terminal end of PR-B that is absent from PR-A, the PR isoforms are identical but their actions are divergent (Kastner *et al*, 1990). PR-B acts as a transcription activator whereas PR-A inhibits PR-B (and other members of the nuclear receptor superfamily) (Vegeto *et al*, 1993).

The PGR has long been hypothesised as a candidate gene for ovarian cancer susceptibility and its variation has been widely studied. Originally, an ALU in intron 7 named PROGINS was identified and found to be associated with increased risk of ovarian cancer (McKenna *et al*, 1995; Rowe *et al*, 1995). Subsequent characterisation of the coding region of the gene identified a non-synonymous single nucleotide polymorphism (SNP) in exon 4 and a synonymous SNP in exon 5 that were in perfect linkage disequilibrium with the PROGINS (De Vivo *et al*, 2002). The PROGINS (or variants in which it is in perfect linkage disequilibrium) has been studied by many groups in relation to ovarian cancer risk. The results are, however, equivocal (McKenna *et al*, 1995; Manolitsas *et al*, 1997; Lancaster *et al*, 1998, 2003; Spurdle *et al*, 2001; Tong *et al*, 2001; Agoulnik *et al*, 2004; Pearce *et al*, 2005; Terry *et al*, 2005; Romano *et al*, 2006). Pearce *et al* (2005), suggested that a variant 3′ of the PGR (rs608995), in partial linkage disequilibrium with the PROGINS, might be a better marker of ovarian cancer risk, but this has not been confirmed by other investigators.

In addition, a putative functional SNP, +331C/T (sometimes denoted as +331G/A), in the promoter region of the PGR that may affect the relative transcription of the PR-A and PR-B isoforms has been found to be associated with a reduced risk of ovarian cancer in studies from North Carolina and Australia (Berchuck *et al*, 2004). This association was particularly strong among clear cell/endometrioid subtypes. However, Risch *et al* (2006) observed an increased risk of ovarian cancer associated with this SNP.

The inconsistent results with the PROGINS and the +331C/T SNP are not surprising. Genetic association studies are plagued by conflicting results that can be explained by heterogeneity across study populations as well as false-positive and -negative results. Lohmueller *et al* (2003) demonstrated that approximately two-thirds of genetic associations do not hold up on meta-analysis. Large sample sizes and pooling of data are therefore critical to evaluate the association between a phenotype and genetic variation with confidence.

To clarify the association between variation at the PGR locus and ovarian cancer risk, including histological subtype associations, 12 groups from the Ovarian Cancer Association Consortium (OCAC) have pooled their data to examine the +331C/T variant (rs10895068), the PROGINS allele (measured by the exon 4 non-synonymous SNP; rs1042838) and a variant 3′ of the PGR (rs608995) in relation to ovarian cancer risk. The results are reported here.

## MATERIALS AND METHODS

### Approval and consent

All study participants provided written informed consent prior to the collection of biological samples or interview/clinical data. Each group involved in the OCAC has Institutional Review Board/ethics approval for this analysis and the University of Southern California and Duke University have Institutional Review Board approval to serve as data coordinating centres for the OCAC.

### Study populations

The OCAC comprises investigators who collaborate on promising genetic associations by combining data from their individual ovarian cancer case–control studies. The participating groups for this PGR study are the Australian Cancer Study, (Merritt *et al*, 2008) the Australian Ovarian Cancer Study (Merritt *et al*, 2008), the Connecticut Ovary Study (CONN) (Risch *et al*, 2006), the Family Registry for Ovarian Cancer Study (Auranen *et al*, 2005; Song *et al*, 2006), the Hormones and Ovarian Cancer Prediction Study, the Danish Malignant Ovarian Cancer Study (MALOVA) (Auranen *et al*, 2005; Song *et al*, 2006), the Mayo Clinic Ovarian Cancer Case–Control Study (Sellers *et al*, 2005), the North Carolina Ovarian Cancer Study (Berchuck *et al*, 2004), the New England-based Case–Control Study (NECC) (Terry *et al*, 2005), the Polish Ovarian Cancer Study (POCS) (García-Closas *et al*, 2007), the UK SEARCH Ovarian Cancer Study (SEARCH) (Auranen *et al*, 2005; Song *et al*, 2006) and the USC/Los Angeles County Case–Control Studies of Ovarian Cancer (USC) (Pearce *et al*, 2005). Details of these studies have been published previously (Gayther *et al*, 2007); Table 1 shows the basic information for each study. The cases analysed here are restricted to women diagnosed with invasive epithelial ovarian cancer.

### Genotyping and quality control

The three SNPs genotyped in this study were the +331C/T (rs10895068), PROGINS (measured by the exon 4 SNP rs1042838) and rs608995 (a variant 3′ of the PGR). The allele designations are based on the forward strand as given in the University of California at Santa Cruz genome browser.

All groups used the 5′ nuclease Taqman allelic discrimination assay (Taqman; Applied Biosystems, Foster City, CA, USA) to genotype samples with the exception of the Australian Cancer Study and Australian Ovarian Cancer Study, which used the iPlex Sequenom MassArray system (Sequenom Inc., San Diego, CA, USA), CONN that used dot blotting (Risch *et al*, 2006), and Mayo Clinic Ovarian Cancer Case–Control Study that used Pyrosequencing for PROGINS and rs608995.

To confirm that laboratory to laboratory quality control was adequate, five SNPs were genotyped in the HAPMAPPT01 panel of CEPH-Utah trios-standard plate provided by Coriell (http://locus.umdny.edu/nigms/nigms_cgi/panel.cgi?id=2&query=HAPMAP01). This 96-well plate contains 90 different DNA samples, five duplicate samples, and a negative template control. Genotyping call rates and concordance between studies were compared. Call rates for these five SNPs ranged from 96 to 99% and the concordance of results across the laboratories was >99%.

Hardy–Weinberg Equilibrium (HWE) was checked among controls by the racial/ethnic group. Data from one study (CONN) for two of the SNPs (PROGINS and rs608995) were excluded for gross deviations (*P*<10^−4^) from HWE. The genotyping calls for studies with minor deviations from HWE (0.01<*P*<0.05) were examined to monitor the quality of the genotyping. There were no obvious reasons for deviation from HWE (e.g., genotyping irregularities), and therefore the minor deviations were assumed most likely due to chance. In addition, results were unchanged when excluding those studies with HWE *P*-values between 0.01 and 0.05 (data not shown). Concordance between duplicate samples was 100% across all studies for the three variants for all data included in these analyses.

Results were available for 12 groups for the +331C/T variant. The NECC study did not genotype rs608995. Results for the PROGINS and rs608995 were excluded for CONN due to significant deviations from HWE. Therefore, results were available for 11 studies for the PROGINS and 10 studies for the rs608995 3′ variant.

### Statistical analysis

The variables available for this analysis were study, race/ethnicity (White, Latina, African-American), age, stage of disease (FIGO), histology (serous, mucinous, clear cell, and endometrioid), and time from diagnosis to blood collection (cases only).

Unconditional logistic regression was used to model the association between each SNP and risk of ovarian cancer stratified on study, age, and race/ethnicity. All single SNP models were log additive. Goodness of fit *P*-values were calculated to evaluate heterogeneity across the study populations. Statistical analyses were carried out using both SAS (Version 9, Cary, NC, USA) and STATA (Version 9, StataCorp, College Station TX, USA). All statistical significance levels (*P*-values) quoted are two-sided. All odds ratios are expressed per copy of the minor allele.

## RESULTS

A total of 4788 invasive epithelial ovarian cancer cases and 7614 controls were available for the current analysis (Table 1). Overall, 92.0% of cases and 93.0% of controls were White and the mean ages were 56.7 and 54.3 years respectively. Information on stage at diagnosis was available on 73.7% of cases, the majority of which were FIGO stage III/IV (63.0%) and 55.5% had a serous histology (Supplementary Table 1).

**Figure 1:**
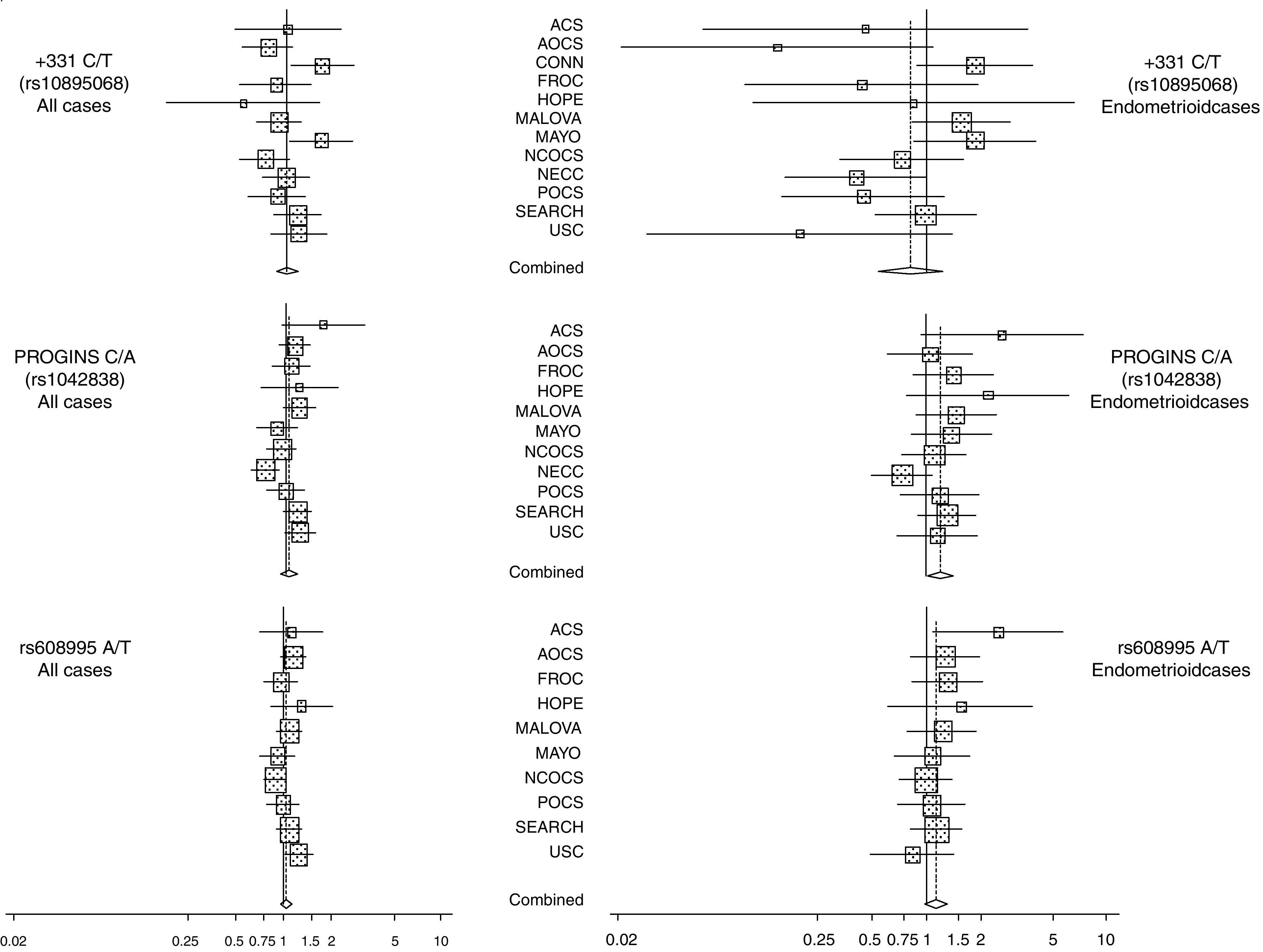
Each panel shows the study-specific and summary odds ratios (boxes) and 95% confidence intervals (lines) for all cases and endometrioid subtype specific results for the three PGR SNPs. The size each box is proportionate to the number of subjects genotyped. See methods for full study names.

Across the studies, the minor allele frequencies in White controls ranged from 4.6 to 7.3% for +331C/T (rs10895068), 9.2 to 19.0% for the PROGINS (rs1042838) and 20.0 to 26.6% for the 3′ variant (rs608995; Supplementary Table 2). The study-specific and summary effect estimates are shown in Figure 1 for all cases and endometrioid subtype associations.

There was no association with the +331C/T (rs10895068) variant among all cases (per allele OR=1.00; 95% CI 0.89–1.13; *P*=1.0; Table 2a). In cell type-specific subgroup analyses, a suggestive association was observed with carrying a T allele and risk of endometrioid invasive ovarian cancers (per allele OR=0.80; 95% CI 0.62–1.04; *P*=0.100; Figure 1). Risk of clear cell ovarian cancer with this variant was reduced to a similar degree (OR=0.83; Table 2b). No associations were observed between serous or mucinous subtypes and this allele (Table 2b).

No overall association was observed with risk of ovarian cancer and the PROGINS allele (rs1042838; OR=1.04; 95% CI 0.96–1.12; *P*=0.38; Table 2a). However, risk was statistically significantly elevated among endometrioid ovarian cancer cases (OR=1.17, 95% CI 1.01–1.36, *P*=0.036; Table 2b).

In a joint effects analysis, risk of endometrioid ovarian cancer associated with the PROGINS was observed only among non-carriers of the +331 minor allele (OR=1.22, 95% CI 1.01–1.46, *P*=0.037). Although not statistically significant, the protective effect of the +331 minor allele persisted among non-carriers of the PROGINS (OR=0.76, 95% CI 0.55–1.06, *P*=0.11) and carriers of the PROGINS (OR=0.79, 95% CI 0.40–1.57, *P*=0.50).

No statistically significant association was observed between the 3′ variant (rs608995) and risk of ovarian cancer when all cases were considered (Table 2a). In subtype analysis, a borderline statistically significant association was observed between endometrioid cases and the rs608995 variant (OR=1.14, 95% CI 0.99–1.31, *P*=0.076), however, this effect was limited to individuals also carrying at least one copy of the PROGINS (data not shown).

## DISCUSSION

Since the publication of the first paper examining the relationship between the PROGINS and ovarian cancer risk more than 10 years ago, there has been substantial interest in the role of the PGR in risk of this disease. We have evaluated three SNPs, +331C/T (rs10895068), PROGINS (rs1042838), and a 3′ variant (rs608995), in the PGR in a pooled ovarian cancer dataset from 12 groups around the world and have found no overall role for this gene in disease risk.

The pooled analysis does provide statistically significant evidence of an association between the PROGINS and risk of invasive endometrioid ovarian cancer. The restriction of an association to this subtype only provides an explanation for the equivocal nature of the published results on the PROGINS and ovarian cancer risk, given that the proportion of endometrioid ovarian cancer cases likely varied by published study and typically accounts for no more than 15–20% of cases.

We also found suggestive evidence of an association between endometrioid ovarian cancer and the +331C/T variant (OR=0.80, 95% CI 0.62–1.04, *P*=0.100). As suggested by Berchuck *et al* (2004), combining endometrioid and clear cell histologies in which the effect is similar, resulted in a borderline statistically significant association (*n*=1088 cases, OR=0.81, 95% CI 0.65–1.01, *P*=0.058).

Pearce *et al* (2005) had previously suggested that rs608995 may explain the PROGINS-ovarian cancer association, however, in this larger dataset in which the effect was restricted to endometrioid cases, this was not supported. When examining the joint effects of the PROGINS and rs608995, the OR for endometrioid ovarian cancer associated with the rs608995 minor allele was 0.79 (95% CI 0.59–1.07, *P*=0.12) in the absence of the PROGINS allele. This suggests that the PROGINS allele or a marker in linkage disequilibrium with the PROGINS is responsible for the association and not the rs608995 variant.

Both the +331 variant and the PROGINS have been studied with regard to their functional effect. The T allele of the +331 favours an increase in the transcription of PR-B relative to PR-A (De Vivo *et al*, 2002); PR-B acts as a classic steroid receptor whereas PR-A acts as a repressor of both PR-B and other steroid receptors. PR-A therefore may lessen overall progesterone responsiveness through its repressive effect. Any variation which increases PR-B relative to PR-A may reduce risk of ovarian cancer by increasing exposure to the beneficial effects of progesterone. In a small study of 107 ovarian cancer cases, decreased risk of death was observed among cases positive for PRB (labelling index>10) relative to cases negative for PRB (*P*=0.037). However, this finding was amongst all cell types (Akahira *et al*, 2000). There is also a suggestion that the PROGINS allele as defined by the V660L exon five variant (as examined in the present study) decreases overall response to progesterone which would be consistent with an increased risk of disease associated with this variant (Romano *et al*, 2007).

In this collaborative effort, there were 4788 ovarian cancer cases, of which 766 (16.0%) were endometrioid tumours. With the samples sizes available in this current OCAC study, we had 80% power to detect odds ratios of 0.83 and 1.12 for the +331, and PROGINS variants, respectively for all cases using a log additive genetic model and a two-sided *α* of 0.05. Among endometrioid subtypes, we had 80% power to detect odds ratios of 0.67 and 1.25 for the +331 and PROGINS variants, respectively using a log additive genetic model and a two-sided *α* of 0.05. Although the power in the current OCAC study is still quite limited, it underscores the importance of collaborative efforts, as the largest individual OCAC study had only 124 endometrioid ovarian cancers. Thus the power of subgroups analyses is clear and will be enhanced in the future with continued patient accrual to existing studies and additional investigators contributing to OCAC studies.

Alternatively, the findings of an association with the +331C/T and PROGINS, variants with the endometrioid histology may simply be due to chance. By assigning priors of 0.05, 0.10, and 0.15, the resulting false positive report probabilities (Wacholder *et al*, 2004) are approximately 0.78, 0.62, and 0.52 for the +331C/T variant and 0.61, 0.42, and 0.32 for the PROGINS, respectively. Thus they may represent false positive findings.

Our analysis is the largest report describing the association between ovarian cancer risk and variants in the PGR. However, there remain several limitations to the study. For example, it is possible that environmental modifiers, such as oral contraceptive use, may be important in refining the PGR ovarian cancer risk associations and such analyses are planned in the future. There are also weaknesses of this study. Firstly, there are variable participation rates for cases between studies (Table 1). If any or all of the variants analysed is related to survival, then the low participation rates among cases might be expected to influence the results. Efforts to evaluate this include the analyses of data stratified by FIGO stage and time from diagnosis to blood collection. None of the results differed significantly when conducting these analyses. Second, as is the nature of collaborative projects, each study had a different level of pathology review and random misclassification cannot be ruled out, which would bias results towards the null in histologic-specific analyses suggesting that our results may be attenuated. Lastly, while we evaluated the best PGR candidate variants suggested by the literature, it remains possible that other, as yet unidentified variants at the locus, influence ovarian cancer risk.

Also, we observed significant heterogeneity of effect for the PROGINS allele and risk of ovarian cancer overall. Evaluation of the heterogeneity by removing one study at a time revealed that the NECC study population had a significantly different odds ratio (OR=0.75, heterogeneity *P*=0.011) from the other 10 OCAC studies. We investigated possible explanations for the heterogeneity we observed in the NECC study, but the reason could not be elucidated. Genotyping error is the most likely reason for experimental bias towards the null. Therefore, we regenotyped the PROGINS allele in the NECC case–control study. The results were 98% concordant with the original genotyping data, ruling this out as an explanation. Also, standard epidemiological risk and protective factors are observed with the NECC study suggesting no coding errors in the data with respect to case–control status. Further stratification of White race by Jewish ancestry was done and the results were consistent across Jewish and non-Jewish Whites (data not shown). The age distribution and participation rates are consistent with the other OCAC studies (Table 1). This heterogeneity may simply be due to chance.

Heterogeneity was also present with the +331 variant and endometrioid ovarian cancer, however no single study accounted for this heterogeneity. The minor allele frequency of this SNP is approximately 5% and the fluctuations in the data may simply represent chance; further follow-up is needed.

If these are true results and variation at the PGR locus is associated with endometrioid ovarian cancer only, then it has implications for the identification of moderate risk genes for ovarian cancer. In the past, ovarian cancer has frequently been treated as a single-disease entity for genetic association studies, mainly because studies have been too small to perform subtype analyses that are substantially powered. However, there is a large body of evidence that indicates different germline and somatic genetic factors contribute to different histological subtypes of ovarian cancer. For example, BRCA1 mutation carriers appear to predispose to serous ovarian cancers (Pal *et al*, 2005); mutations in the PTEN tumour suppressor gene are more associated with endometrioid ovarian cancers (Obata *et al*, 1998); and K-ras mutations are more common in mucinous tumours than in either serous of endometrioid subtypes (Gemignani *et al*, 2003).

In conclusion, in the present analysis, we were able to exclude an overall effect of these variants in the PGR with risk of ovarian cancer. However, our evidence suggests histology-specific effects, demonstrating the necessity of data pooling to examine subgroup effects for this cancer. Although the PROGINS is unlikely to represent appreciable susceptibility risk factor, given the restriction of the association to endometrioid histology, the magnitude of the observed odds ratio, and the modest allele frequency of this variant, further analysis of this gene with regard to the endometrioid subtype is warranted to provide insight into the mechanisms underlying disease aetiology.

## Figures and Tables

**Table 1:** Characteristics of the 12 case–control studies used in this analysis

	**Cases**				**Controls**				
**Study^a^**	**Ascertainment**	***N***	**White (%)**	**Age (mean)**	**Ascertainment**	***N***	**White (%)**	**Age (mean)**	**Participation rates**
ACS, Australia	Cancer registries of New South Wales and Victoria: cases diagnosed July 2002–June 2005.	111	91.0	59.8	Randomly selected from Commonwealth electoral roll. Frequency matched for age and geographical region	156	95.8	55.2	
AOCS, Australia	Diagnosed from 2002 onwards; recruited through surgical treatment centres throughout Australia and cancer registries of Queensland, southern Australia and western Australia cases diagnosed 2002–2006.	502	95.4	59.7	Randomly selected from Commonwealth electoral roll. Frequency matched for age and geographical region	684	97.4	58.2	Cases: 68% Controls: 47%
CONN, USA	Rapid case ascertainment of consecutive cases identified from 30 Connecticut hospitals and through the Connecticut Tumour Registry between 1998 and 2003	365	90.7	59.1	HCFA (CMS) plus random-digit dial identification from study area, frequency matched to cases on age group	533	88.6	53.1	Cases: 69% Controls 61%
FROC, USA	Consecutive cases diagnosed from 1997–2002 in Greater Bay Area Cancer Registry, San Francisco.	324	87.3	50.8	Random-digit dial identification from study area. Frequency matched to cases for race/ethnicity and 5-year age group	424	86.8	48.4	Cases: 75% Controls: 91%
HOPE, USA	Variable source including physician offices, cancer registries and pathology databases from counties of western Pennsylvania, eastern Ohio and western New York.	57	95.1	57.9	Identified in same regions. Frequency matched for age and ethnicity. All participants undergo home interviews	152	94.7	56.1	Cases: 69% Controls: 81%
MALOVA, Denmark	Incident cases (35–79 years) diagnosed 1994–1999 from municipalities of Copenhagen and Frederiksberg and surrounding counties.	444	100.0	59.9	Random sample of general female population (35–79 years) in study area, selected using computerised Central Population Register, matched to cases for age and geographical region	1221	100	56.8	Cases: 79% Controls: 67%
MAYO, USA	Cases attending Mayo Clinic diagnosed from 2000 onwards, identified in a six-state surrounding region.	278	97.6	61.4	Identified through Mayo Clinic. Healthy women seeking general medical examination. Frequency matched to cases for age, race, and state of residence	389	97.7	60.3	Cases: 84% Controls: 65%
NCOCS, USA	Cases from 1999 onwards, identified from 48 counties within the region by rapid-case ascertainment.	610	83.0	56.8	Controls identified from same region. Frequency matched to cases for age and race	843	81.5	54.4	Cases: 70% Controls: 63%
NECC, USA	Cases identified through hospital tumour boards and state cancer registries in New Hampshire and Massachusetts from 1992 to 2003.	667	96.0	53.6	Controls identified through a combination of random-digit dialling, town books, and drivers' license lists and frequency matched to cases on age and state of residence	1011	96.6	50.5	Cases: 72% Controls: 69%
POCS, Poland	Cases collected from cities of Warsaw and Lodz, 2001–2003, by rapid ascertainment at participating hospitals	264	100.0	56.3	Identified at random through The Polish Electronic System. Stratified by city and 5-year age categories	625	100	56.1	Cases: 71% Controls: 67%
SEARCH, UK	Cases <70 years from East Anglian, West Midlands and Trent regions of England. Prevalent cases diagnosed 1991–1998; incident cases diagnosed 1998 onwards.	643	99.3	55.8	Selected from the EPIC-Norfolk cohort of 25 000 individuals aged 45–74, based in the same geographical regions as the cases	852	99.7	52.7	Cases: 67% Controls: 84%
USC, USA	Rapid case ascertainment through Los Angeles Cancer Surveillance Program from 1993 onwards	523	71.0	54.9	Neighborhood recruited controls, frequency matched to cases for age and ethnicity	724	75.4	52.7	Cases: 73% Controls: 73%

a See methods for full study name.

**Table 2a:** Summary odds ratios (per allele) and 95% CI for the three PGR SNPs for all invasive cases among OCAC studies

	**Controls**		**All cases**	
**SNP**	***N***	***N***	**OR^a^ (95% CI)**	***P***
+331C/T (rs10895068)	7338	4551	1.00 (0.89–1.13)	1.0
PROGINS C/A (rs1042838)	6794	4124	1.04 (0.96–1.12)	0.38
rs608995 A/T	5796	3510	1.05 (0.98–1.13)	0.17

CI=confidence interval; OR=odds ratio; OCAC=Ovarian Cancer Association Consortium; PGR=progesterone receptor gene; SNP=single nucleotide polymorphism.

a All analyses stratified on study, race, and age.

**Table 2b:** Summary odds ratios (per allele) and 95% CI for the three PGR SNPs by histology among OCAC studies

	**Clear cell**			**Endometrioid**			**Mucinous cases**			**Serous cases**		
**SNP**	***N***	**OR^a^ (95% CI)**	***P***	***N***	**OR^a^ (95% CI)**	***P***	***N***	**OR^a^ (95% CI)**	***P***	***N***	**OR^a^ (95% CI)**	***P***
+331C/T (rs10895068)	363	0.83 (0.58–1.19)	0.31	725	0.80 (0.62–1.04)	0.100	321	0.98 (0.68–1.40)	0.90	2549	1.06 (0.92–1.22)	0.44
PROGINS C/A (rs1042838)	324	0.98 (0.79–1.22)	0.88	651	1.17 (1.01–1.36)	0.036	296	1.04 (0.83–1.30)	0.76	2285	0.99 (0.90–1.08)	0.77
rs608995 A/T	252	1.00 (0.81–1.23)	0.98	528	1.14 (0.99–1.31)	0.076	262	1.09 (0.89–1.34)	0.41	1966	1.03 (0.94-1.12)	0.53

CI=confidence interval; OR=odds ratio; OCAC=Ovarian Cancer Association Consortium; PGR=progesterone receptor gene; SNP=single nucleotide polymorphism; N=number of cases.

a All analyses stratified on study, race and age.

## References

[bib1] Agoulnik IU, Tong XW, Fischer DC, Korner K, Atkinson NE, Edwards DP, Headon DR, Weigel NL, Kieback DG (2004) A germline variation in the progesterone receptor gene increases transcriptional activity and may modify ovarian cancer risk. J Clin Endocrinol Metab 89: 6340–6347 15579801 10.1210/jc.2004-0114

[bib2] Akahira J, Inoue T, Suzuki T, Ito K, Konno R, Sato S, Moriya T, Okamura K, Yajima A, Sasano H (2000) Progesterone receptor isoforms A and B in human epithelial ovarian carcinoma: immunohistochemical and RT-PCR studies. Br J Cancer 83: 1488–1494 11076658 10.1054/bjoc.2000.1463 PMC2363436

[bib3] Auranen A, Song H, Waterfall C, Dicioccio RA, Kuschel B, Kjaer SK, Hogdall E, Hogdall C, Stratton J, Whittemore AS, Easton DF, Ponder BA, Novik KL, Dunning AM, Gayther S, Pharoah PD (2005) Polymorphisms in DNA repair genes and epithelial ovarian cancer risk. Int J Cancer 117: 611–618 15924337 10.1002/ijc.21047

[bib4] Berchuck A, Schildkraut JM, Wenham RM, Calingaert B, Ali S, Henriott A, Halabi S, Rodriguez GC, Gertig D, Purdie DM, Kelemen L, Spurdle AB, Marks J, Chenevix-Trench G (2004) Progesterone receptor promoter +331A polymorphism is associated with a reduced risk of endometrioid and clear cell ovarian cancers. Cancer Epidemiol Biomarkers Prev 13: 2141–2147 15598772

[bib5] Cooper GS, Schildkraut JM, Whittemore AS, Marchbanks PA (1999) Pregnancy recency and risk of ovarian cancer. Cancer Causes Control 10: 397–402 10530609 10.1023/a:1008960520316

[bib6] De Vivo I, Huggins GS, Hankinson SE, Lescault PJ, Boezen M, Colditz GA, Hunter DJ (2002) A functional polymorphism in the promoter of the progesterone receptor gene associated with endometrial cancer risk. Proc Natl Acad Sci USA 99: 12263–12268 12218173 10.1073/pnas.192172299 PMC129433

[bib7] García-Closas M, Brinton LA, Lissowska J, Sherman ME, Szeszenia-Dabrowska N, Peplonska B, Welch R, Yeager M, Bardin-Mikolajczak A, Zatonski W, Chanock SJ (2007) Ovarian cancer risk and common variation in the sex hormone-binding globulin gene: a population-based case–control study. BMC Cancer 7: 60 17411440 10.1186/1471-2407-7-60 PMC1855931

[bib8] Gayther SA, Song H, Ramus SJ, Kjaer SK, Whittemore AS, Quaye L, Tyrer J, Shadforth D, Hogdall E, Hogdall C, Blaeker J, DiCioccio R, McGuire V, Webb PM, Beesley J, Green AC, Whiteman DC, Australian Ovarian Cancer Study Group, Goodman MT, Lurie G, Carney ME, Modugno F, Ness RB, Edwards RP, Moysich KB, Goode EL, Couch FJ, Cunningham JM, Sellers TA, Wu AH, Pike MC, Iversen ES, Marks JR, Garcia-Closas M, Brinton L, Lissowska J, Peplonska B, Easton DF, Jacobs I, Ponder BA, Schildkraut J, Pearce CL, Chenevix-Trench G, Berchuck A, Pharoah PDP, Ovarian Cancer Association Consortium (2007) Tagging single nucleotide polymorphisms in cell cycle control genes and susceptibility to invasive epithelial ovarian cancer. Cancer Res 67: 3027–3035 17409409 10.1158/0008-5472.CAN-06-3261

[bib9] Gemignani ML, Schlaerth AC, Bogomolniy F, Barakat RR, Lin O, Soslow R, Venkatraman E, Boyd J (2003) Role of KRAS and BRAF gene mutations in mucinous ovarian carcinoma. Gynecol Oncol 90: 378–381 12893203 10.1016/s0090-8258(03)00264-6

[bib10] Hartge P, Schiffman MH, Hoover R, McGowan L, Lesher L, Norris HJ (1989) A case–control study of epithelial ovarian cancer. Am J Obstet Gynecol 161: 10–16 2750791 10.1016/0002-9378(89)90221-4

[bib11] Kastner P, Krust A, Turcotte B, Stropp U, Tora L, Gronemeyer H, Chambon P (1990) Two distinct estrogen-regulated promoters generate transcripts encoding the two functionally different human progesterone receptor forms A and B. EMBO J 9: 1603–1614 2328727 10.1002/j.1460-2075.1990.tb08280.x PMC551856

[bib12] Lancaster JM, Berchuck A, Carney ME, Wiseman R, Taylor JA (1998) Progesterone receptor gene polymorphism and risk for breast and ovarian cancer. Br J Cancer 78: 277 10.1038/bjc.1998.480 PMC2062885 9683307

[bib13] Lancaster JM, Wenham RM, Halabi S, Calingaert B, Marks JR, Moorman PG, Bentley RC, Berchuck A, Schildkraut JM (2003) No relationship between ovarian cancer risk and progesterone receptor gene polymorphism in a population-based, case–control study in North Carolina. Cancer Epidemiol Biomarkers Prev 12: 226–227 12646513

[bib14] Lohmueller KE, Pearce CL, Pike M, Lander ES, Hirschhorn JN (2003) Meta-analysis of genetic association studies supports a contribution of common variants to susceptibility to common disease. Nat Genet 33: 177–182 12524541 10.1038/ng1071

[bib15] Manolitsas TP, Englefield P, Eccles DM, Campbell IG (1997) No association of a 306-bp insertion polymorphism in the progesterone receptor gene with ovarian and breast cancer. Br J Cancer 75: 1398–1399 9155067 10.1038/bjc.1997.238 PMC2228221

[bib16] Merritt MA, Green AC, Nagle CM, Webb PM, Australian Cancer Study (Ovarian Cancer), Australian Ovarian Cancer Study Group (2008) Talcum powder, chronic pelvic inflammation and NSAIDs in relation to risk of epithelial ovarian cancer. Int J Cancer 122: 170–176 17721999 10.1002/ijc.23017

[bib17] McKenna NJ, Kieback DG, Carney DN, Fanning M, McLinden J, Headon DR (1995) A germline TaqI restriction fragment length polymorphism in the progesterone receptor gene in ovarian carcinoma. Br J Cancer 71: 451–455 7880723 10.1038/bjc.1995.92 PMC2033643

[bib18] Ness RB, Grisso JA, Klapper J, Schlesselman JJ, Silberzweig S, Vergona R, Morgan M, Wheeler JE (2000) Risk of ovarian cancer in relation to estrogen and progestin dose and use characteristics of oral contraceptives. SHARE study group. Steroid Hormones and reproductions. Am J Epidemiol 152: 233–241 10933270 10.1093/aje/152.3.233

[bib19] Norman AW, Litwack G (1997) Estrogens and progestins. In Hormones Norman AW, Litwack G (eds), pp 361–386. San Diego, CA: Academic Press

[bib20] Obata K, Morland SJ, Watson RH, Hitchcock A, Chenevix-Trench G, Thomas EJ, Campbell IG (1998) Frequent PTEN/MMAC mutations in endometrioid but not serous or mucinous epithelial ovarian tumors. Cancer Res 58: 2095–2097 9605750

[bib21] Pal T, Permuth-Wey J, Betts JA, Krischer JP, Fiorica J, Arango H, LaPolla J, Hoffman M, Martino MA, Wakeley K, Wilbanks G, Nicosia S, Cantor A, Sutphen R (2005) BRCA1 and BRCA2 mutations account for a large proportion of ovarian carcinoma cases. Cancer 104: 2807–2816 16284991 10.1002/cncr.21536

[bib22] Pearce CL, Hirschhorn JN, Wu AH, Burtt NP, Stram DO, Young S, Kolonel LN, Henderson BE, Altshuler D, Pike MC (2005) Clarifying the PROGINS allele association in ovarian and breast cancer risk: a haplotype-based analysis. J Natl Cancer Inst 97: 51–59 15632380 10.1093/jnci/dji007

[bib23] Pike MC, Pearce CL, Peters R, Cozen W, Wan P, Wu AH (2004) Hormonal factors and the risk of invasive ovarian cancer: a population-based case–control study. Fertil Steril 82: 186–195 15237010 10.1016/j.fertnstert.2004.03.013

[bib24] Risch HA (1998) Hormonal etiology of epithelial ovarian cancer, with a hypothesis concerning the role of androgens and progesterone. J Natl Cancer Inst 90: 1774–1786 9839517 10.1093/jnci/90.23.1774

[bib25] Risch HA, Bale AE, Beck PA, Zheng W (2006) PGR +331 A/G and increased risk of epithelial ovarian cancer. Cancer Epidemiol Biomarkers Prev 15: 1738–1741 16985038 10.1158/1055-9965.EPI-06-0272

[bib26] Rodriguez GC, Walmer DK, Cline M, Krigman H, Lessey BA, Whitaker RS, Dodge R, Hughes CL (1998) Effect of progestin on the ovarian epithelium of macaques: cancer prevention through apoptosis? J Soc Gynecol Investig 5: 271–276 10.1016/s1071-5576(98)00017-3 9773403

[bib27] Romano A, Delvoux B, Fischer DC, Groothuis P (2007) The PROGINS polymorphism of the human progesterone receptor diminishes the response to progesterone. J Mol Endocrinol 38: 331–350 17293450 10.1677/jme.1.02170

[bib28] Romano A, Lindsey PJ, Fischer DC, Delvoux B, Paulussen AD, Janssen RG, Kieback DG (2006) Two functionally relevant polymorphisms in the human progesterone receptor gene (+331 G/A and progins) and the predisposition for breast and/or ovarian cancer. Gynecol Oncol 101: 287–295 16360811 10.1016/j.ygyno.2005.10.040

[bib29] Rosenberg L, Palmer JR, Zauber AG, Warshauer ME, Lewis Jr JL, Strom BL, Harlap S, Shapiro S (1994) A case–control study of oral contraceptive use and invasive epithelial ovarian cancer. Am J Epidemiol 139: 654–661 8166126 10.1093/oxfordjournals.aje.a117055

[bib30] Rosenblatt KA, Thomas DB, Noonan EA (1992) High-dose and low-dose combined oral contraceptives: protection against epithelial ovarian cancer and the length of the protective effect. The WHO Collaborative Study of Neoplasia and Steroid Contraceptives. Eur J Cancer 28A: 1872–1876 1389530 10.1016/0959-8049(92)90026-x

[bib31] Rowe SM, Coughlan SJ, McKenna NJ, Garrett E, Kieback DG, Carney DN, Headon DR (1995) Ovarian carcinoma-associated TaqI restriction fragment length polymorphism in intron G of the progesterone receptor gene is due to an Alu sequence insertion. Cancer Res 55: 2743–2745 7796397

[bib32] Royar J, Becher H, Chang-Claude J (2001) Low-dose oral contraceptives: protective effect on ovarian cancer risk. Int J Cancer 95: 370–374 11668519 10.1002/1097-0215(20011120)95:6<370::aid-ijc1065>3.0.co;2-t

[bib33] Schildkraut JM, Calingaert B, Marchbanks PA, Moorman PG, Rodriguez GC (2002) Impact of progestin and estrogen potency in oral contraceptives on ovarian cancer risk. J Natl Cancer Inst 94: 32–38 11773280 10.1093/jnci/94.1.32

[bib34] Sellers TA, Schildkraut JM, Pankratz VS, Vierkant RA, Fredericksen ZS, Olson JE, Cunningham J, Taylor W, Liebow M, McPherson C, Hartmann LC, Pal T, Adjei AA (2005) Estrogen bioactivation, genetic polymorphisms, and ovarian cancer. Cancer Epidemiol Biomarkers Prev 14: 2536–2543 16284375 10.1158/1055-9965.EPI-05-0142

[bib35] Song H, Ramus SJ, Quaye L, DiCioccio RA, Tyrer J, Lomas E, Shadforth D, Hogdall E, Hogdall C, McGuire V, Whittemore AS, Easton DF, Ponder BA, Kjaer SK, Pharoah PD, Gayther SA (2006) Common variants in mismatch repair genes and risk of invasive ovarian cancer. Carcinogenesis 27: 2235–2242 16774946 10.1093/carcin/bgl089

[bib36] Spurdle AB, Webb PM, Purdie DM, Chen X, Green A, Chenevix-Trench G (2001) No significant association between progesterone receptor exon 4 Val660Leu G/T polymorphism and risk of ovarian cancer. Carcinogenesis 22: 717–721 11323389 10.1093/carcin/22.5.717

[bib37] Study C (1987) The reduction in risk of ovarian cancer associated with oral-contraceptive use. The cancer and steroid hormone study of the centers for disease control and the national institute of child health and human development. N Engl J Med 316: 650–655 3821795 10.1056/NEJM198703123161102

[bib38] Terry KL, De Vivo I, Titus-Ernstoff L, Sluss PM, Cramer DW (2005) Genetic variation in the progesterone receptor gene and ovarian cancer risk. Am J Epidemiol 161: 442–451 15718480 10.1093/aje/kwi064 PMC1380205

[bib39] Titus-Ernstoff L, Perez K, Cramer DW, Harlow BL, Baron JA, Greenberg ER (2001) Menstrual and reproductive factors in relation to ovarian cancer risk. Br J Cancer 84: 714–721 11237375 10.1054/bjoc.2000.1596 PMC2363792

[bib40] Tong D, Fabjani G, Heinze G, Obermair A, Leodolter S, Zeillinger R (2001) Analysis of the human progesterone receptor gene polymorphism progins in Austrian ovarian carcinoma patients. Int J Cancer 95: 394–397 11668524 10.1002/1097-0215(20011120)95:6<394::aid-ijc1070>3.0.co;2-x

[bib41] Vegeto E, Shahbaz MM, Wen DX, Goldman ME, O'Malley BW, McDonnell DP (1993) Human progesterone receptor A form is a cell- and promoter-specific repressor of human progesterone receptor B function. Mol Endocrinol 7: 1244–1255 8264658 10.1210/mend.7.10.8264658

[bib42] Wacholder S, Chanock S, Garcia-Closas M, El Ghormli L, Rothman N (2004) Assessing the probability that a positive report is false: an approach for molecular epidemiology studies. J Natl Cancer Inst 96: 434–442 15026468 10.1093/jnci/djh075 PMC7713993

[bib43] Whiteman DC, Siskind V, Purdie DM, Green AC (2003) Timing of pregnancy and the risk of epithelial ovarian cancer. Cancer Epidemiol Biomarkers Prev 12: 42–46 12540502

[bib44] Zhou H, Luo MP, Schonthal AH, Pike MC, Stallcup MR, Blumenthal M, Zheng W, Dubeau L (2002) Effect of reproductive hormones on ovarian epithelial tumors: I. Effect on cell cycle activity. Cancer Biol Ther 1: 300–306 12432283 10.4161/cbt.86

