# Supplementary material for: Progesterone receptor variation and risk of ovarian cancer is limited to the invasive endometrioid subtype: results from the ovarian cancer association consortium pooled analysis
Source: Br J Cancer. 2008 Jan 22;98(2):282–8. doi: 10.1038/sj.bjc.6604170 (PMC2361465; doi:10.1038/sj.bjc.6604170)
Supplement: Supplementary file 1 [file 6604170x1.doc]

Supplementary Table 1. Clinical characteristics of the invasive epithelial ovarian cancer cases (n=4788) by study.

	Stage*			Histology‡				
	% Known	I/II n (%)	III/IV n (%)	Serous n (%)	Endometrioid n (%)	Clear Cell n (%)	Mucinous n (%)	Other n (%)
ACS	28.8	8 (25.0)	24 (75.0)	70 (63.1)	16 (14.4)	12 (10.8)	3 (2.7)	10 (9.0)
AOCS	86.4	113 (26.0)	321 (74.0)	345 (68.7)	55 (11.0)	26 (5.2)	11 (2.2)	65 (13.0)
CONN	0	n/a	n/a	218 (59.7)	74 (20.3)	33 (9.0)	17 (4.7)	23 (6.3)
FROC	94.7	121 (39.4)	186 (60.6)	164 (50.6)	47 (14.5)	23 (7.1)	29 (9.0)	61 (18.8)
HOPE	96.5	22 (40.0)	33 (60.00)	34 (59.7)	9 (15.8)	1 (1.8)	1 (1.8)	12 (21.1)
MALOVA	100.0	147 (33.1)	297 (66.9)	274 (61.7)	56 (12.6)	32 (7.2)	43 (9.7)	39 (8.8)
MAYO	93.2	62 (23.8)	197 (76.3)	182 (65.5)	50 (18.0)	14 (5.0)	7 (2.5)	25 (9.0)
NCOCS	98.0	181 (30.3)	417 (69.7)	354 (58.0)	91 (14.9)	61 (10.0)	31 (5.1)	73 (12.0)
NECC†	0	n/a	n/a	352 (52.8)	124 (18.6)	81 (12.1)	38 (5.7)	72 (10.8)
POCS	67.4	71 (39.9)	107 (60.1)	118 (44.7)	58 (22.0)	14 (5.3)	26 (9.9)	48 (18.2)
SEARCH	67.2	288 (66.7)	144 (33.3)	220 (34.2)	117 (18.2)	58 (9.0)	89 (13.8)	159 (24.7)
USC	99.5	193 (37.0)	328 (63.0)	327 (62.5)	69 (13.2)	36 (6.9)	51 (9.8)	40 (7.7)
ALL	73.7	1206 (37.0)	2054 (63.0)	2658 (55.6)	766 (16.0)	391 (8.2)	346 (7.2)	627 (13.1)

*FIGO stage calculated based on cases with data available.

†Stage data not available

‡Other represents mixed cell type and unclassified invasive epithelial ovarian cancers.
