# Supplementary material for: Progesterone receptor variation and risk of ovarian cancer is limited to the invasive endometrioid subtype: results from the ovarian cancer association consortium pooled analysis
Source: Br J Cancer. 2008 Jan 22;98(2):282–8. doi: 10.1038/sj.bjc.6604170 (PMC2361465; doi:10.1038/sj.bjc.6604170)
Supplement: Supplementary file 2 [file 6604170x2.doc]

Supplementary Table 2. Minor allele frequencies and Hardy-Weinberg equilibrium p-values by SNP and Study for White controls (n=7064).

	+331C/T (rs10895068)		PROGINS C/A (rs1042838)		rs608995 (A/T)	
	T*	HWE p	A*	HWE p	T*	HWE p
ACS	6.6	0.41	9.2	0.24	20.0	0.45
AOCS	7.0	0.64	15.4	0.11	23.9	0.02
CONN	4.6	0.30	na†	<0.0001	na†	<0.0001
FROC	4.9	0.32	15.5	0.10	26.2	0.65
HOPE	6.6	0.62	14.2	0.03	22.7	0.01
MALOVA	6.6	0.32	12.1	0.22	21.0	0.41
MAYO	4.9	0.93	16.5	0.30	25.0	0.97
NCOCS	6.4	0.88	18.2	0.10	26.6	0.05
NECC	4.8	0.38	19.0	0.31	nd‡	nd‡
POCS	7.3	0.45	16.1	0.80	23.7	0.14
SEARCH	5.5	0.34	14.5	0.01	21.9	0.28
USC	4.6	0.07	16.4	0.91	24.0	0.23

*Minor allele frequency

†Data excluded due to significant deviation from HWE

‡SNP not genotyped
